# Race/ethnicity-associated blood DNA methylation differences between Japanese and European American women: an exploratory study

**DOI:** 10.1186/s13148-021-01171-w

**Published:** 2021-10-11

**Authors:** Min-Ae Song, Anna Eames Seffernick, Kellie J. Archer, Kellie M. Mori, Song-Yi Park, Linda Chang, Thomas Ernst, Maarit Tiirikainen, Karolina Peplowska, Lynne R. Wilkens, Loïc Le Marchand, Unhee Lim

**Affiliations:** 1 Division of Environmental Health Science, College of Public Health, The Ohio State University, 404 Cunz Hall, 1841 Neil Ave., Columbus, OH 43210 USA; 2 Division of Biostatistics, College of Public Health, The Ohio State University, Columbus, OH USA; 3 Population Sciences in the Pacific Program, University of Hawaii Cancer Center, University of Hawaii at Manoa, Honolulu, HI USA; 4 School of Medicine, University of Maryland, Baltimore, MD USA; 5 Genomics and Bioinformatics Shared Resources, University of Hawaii Cancer Center, University of Hawaii at Manoa, Honolulu, HI USA

## Abstract

**Background:**

Racial/ethnic disparities in health reflect a combination of genetic and environmental causes, and DNA methylation may be an important mediator. We compared in an exploratory manner the blood DNA methylome of Japanese Americans (JPA) versus European Americans (EUA).

**Methods:**

Genome-wide buffy coat DNA methylation was profiled among healthy Multiethnic Cohort participant women who were Japanese (JPA; n = 30) or European (EUA; n = 28) Americans aged 60–65. Differentially methylated CpGs by race/ethnicity (DM-CpGs) were identified by linear regression (Bonferroni-corrected *P* < 0.1) and analyzed in relation to corresponding gene expression, a priori selected single nucleotide polymorphisms (SNPs), and blood biomarkers of inflammation and metabolism using Pearson or Spearman correlations (FDR < 0.1).

**Results:**

We identified 174 DM-CpGs with the majority of hypermethylated in JPA compared to EUA (n = 133), often in promoter regions (n = 48). Half (51%) of the genes corresponding to the DM-CpGs were involved in liver function and liver disease, and the methylation in nine genes was significantly correlated with gene expression for DM-CpGs. A total of 156 DM-CpGs were associated with rs7489665 (*SH2B1*). Methylation of DM-CpGs was correlated with blood levels of the cytokine MIP1B (n = 146). We confirmed some of the DM-CpGs in the TCGA adjacent non-tumor liver tissue of Asians versus EUA.

**Conclusion:**

We found a number of differentially methylated CpGs in blood DNA between JPA and EUA women with a potential link to liver disease, specific SNPs, and systemic inflammation. These findings may support further research on the role of DNA methylation in mediating some of the higher risk of liver disease among JPA.

**Supplementary Information:**

The online version contains supplementary material available at 10.1186/s13148-021-01171-w.

## Introduction

Racial/ethnic disparities in health are phenotype-specific [1]. While Asian Americans have the longest life expectancy among racial/ethnic populations in the USA based on lower mortality from cardiovascular disease and cancer [2], they are known to have a higher susceptibility to obesity-related metabolic diseases [3]. In the Multiethnic Cohort (MEC) based on five race/ethnic populations in Hawaii and Southern California [4], we have observed that Asian Americans tend to develop metabolic syndrome and related diseases, such as type 2 diabetes, starting at a lower level of body mass index (BMI) compared to other racial/ethnic groups [5]. Also, we observed in a magnetic resonance imaging study in a subset of the MEC that Asian Americans have been observed to accumulate disproportionately higher amounts of visceral fat and liver fat across a wide range of BMI or total body fat mass [6]. Consistently, we and others have reported the highest BMI- or total fat mass-adjusted prevalence of non-alcoholic fatty liver disease (NAFLD) and NAFLD-associated hepatocellular carcinoma among Asian Americans [7–9]. Some of these disparities have been attributed to genetic differences, such as the *PNPLA3* risk variant for NAFLD (rs738409), which is more common in Asians and Latinos [10]. Still, underlying biological mechanisms on racial/ethnic disparities in health are poorly understood [11].

Epigenetics may be key to elucidate the mechanisms underlying the racial/ethnic differences in health and disease [12, 13]. DNA methylation is an epigenetic regulator not determined by the DNA sequence. Unlike DNA sequences, the DNA methylome composition is dynamic and influenced by both genetic and environmental factors [14]. For example, altered blood DNA methylation has been observed to be associated with various cardiometabolic diseases and correlated with changes in target tissue DNA methylation, implicating related cellular processes [15]. However, most studies to date for disease associations have been conducted in European descent individuals, and data comparing blood DNA methylation patterns by race/ethnicity are limited [12]. Global blood DNA methylation, as determined by (3H)-methyl acceptance or LINE-1 repeat element methylation, was described to be lower [16, 17] or higher [18] in non-European compared to European descent individuals. Recent genome-wide methylation array analyses reported differentially methylated CpGs in African Americans versus European Americans [19].

Considering the potentially important role of DNA methylation in mediating the racial/ethnic health disparities as reviewed above, and based on our previous findings of distinct metabolic disparities among Asian Americans for NAFLD and related liver disease in the MEC [5–8, 11], we examined the blood DNA methylome between heathy Japanese American and European American women in the current study. Specifically, we identified differentially methylated CpGs between Japanese Americans and European Americans. Further, we examined how they are associated with corresponding gene expression, related genetic variants, and blood biomarkers of metabolism.

## Materials and methods

### Study participants

Participants for this study were recruited from the Multiethnic Cohort Study (MEC; 1993-current) [4]. As detailed previously [20, 21], in 2009–2019, 60 overall healthy, postmenopausal women aged 60–65 were recruited among the MEC participants in Oahu, Hawaii, including self-identified 30 European Americans (EUA) and 30 Japanese Americans (JPA) selected after stratification on age and BMI. This ancillary study within the MEC was a pilot study to explore imaging-based body composition of JPA, who were observed to have higher risks for obesity-related diseases and cancers in the MEC [5, 7, 8] despite their lower mean BMI compared to EUA among generally healthy individuals. The small pilot study included only women due to limited resources and was designed to compare the body composition of generally healthy JPA versus EUA women with comparable BMIs using BMI-stratified recruitment. The participants underwent a detailed body composition assessment, involving a whole-body dual-energy X-ray absorptiometry (DXA) and an abdominal magnetic resonance imaging (MRI) scan, and provided fasting blood and responses to health-related questionnaires.

### Genome-wide DNA methylation assay and data processing

The Illumina Infinium HumanMethylation450K BeadChips array (Illumina, San Diego, CA) (HM450) was used to profile DNA methylation. Fifty-eight samples (28 EUA and 30 JPA) were available for methylation assays. The QIAamp DNA Mini Kit (Qiagen, Valencia, CA) was used to extract DNA from a fasting blood buffy coat that was stored at − 80 °C. For each sample, 500 ng of DNA was bisulfite converted per the manufacturer’s specifications for the HM450 using the EZ DNA Kit (Zymo Research, Irvine, CA). The bisulfite-converted DNA was then hybridized onto the HM450 according to the Illumina Infinium HD Methylation protocol. Images were generated on the Illumina iScan SQ scanner. GenomeStudio (v.2011.1) Methylation module (v.1.9.0) software was used to extract image intensities.

Raw intensity data from the HM450 (.idat files) were read into R version 3.5.2 [22] using the Bioconductor [23] package minfi [24]. Using the probe intensities, β-values were determined. We used the M-values (logit-transformed β) in our analyses for reduced heteroscedasticity [25]. We started with 485,512 CpGs, and Subset-quantile Within Array Normalization (SWAN) was used to normalize the data [26]. Before further analysis, the data were filtered to remove problematic CpGs. Specifically, Illumina annotation was used to identify CpGs that overlap with single nucleotide polymorphisms (SNPs) or are within 10 base pairs (bp) of SNPs, which were then removed (n = 89,678). We also excluded probes that were in the Y-chromosome, off-target (n = 31,554) [27, 28], or had a detection *P*-value > 0.05. Additionally, CpGs with extremely high methylation (β ≥ 0.9) or low methylation values (β ≤ 0.1) were removed (n = 77,715) for all samples [29] as these CpGs can be considered to be fully methylated or fully unmethylated, respectively [29, 30]. After filtering, 285,457 CpGs remained for analysis. The reference genome for this study was GRCh37/hg19 (Human Genome version 19).

Because the methylation assays were performed in two batches (mixed-race/ethnicity per batch), batch effects were adjusted for using the ComBat function [31] (R, sva [32]). We further adjusted for whole blood cell composition to minimize potential confounding [33, 34]. The proportions of six major cell types in blood DNA (CD8 + T cells, CD4 + T cells, natural killer cells, B cells, monocyte, and granulocyte) were estimated using the estimateCellCounts function (Bioconductor, minfi) [35]. The isometric log-ratio transformation was applied to the matrix of cell compositions, and the transformed values were used as additional covariates in statistical models for associations [36].

### Genome-wide transcriptome

RNA was extracted from the participants’ stored whole blood using the PAXgene Kit (Qiagen), and the quality of the RNA was checked on the Agilent Bioanalyzer (Agilent Technologies, Santa Clara, CA), indicating high integrity, with an average RIN of 8.2 (range 7–9). 100 ng of RNA was used for gene expression analysis by the Affymetrix GeneChip Human Transcriptome Array 2.0 (HTA 2.0; Affymetrix Inc., Santa Clara, CA). An Affymetrix GeneChip scanner 3000 with AGCC Software (Affymetrix GeneChip® Command Console®) was used to scan the arrays. Transcriptome Analysis Console 4.0 (TAC 4.0; Thermo Fisher Scientific, Waltham, MA) was used to assess sample quality, and 2 samples with poor quality were removed, leaving 56 samples for the CpG-expression analysis. The CEL files generated by the arrays were imported into R using the Bioconductor oligo package [37]. The Robust Multi-Array Average procedure was used to normalize the data and obtain probe set expression summaries [38–40]. The data was annotated using the hta20transcriptcluster.db package [41].

Pairs of expression (transcripts) with *cis* methylation (probes) were identified for each of the 56 participants (28 JPA, 28 EUA). The HM450 annotation was used to assign gene names to the CpGs, which were then matched to Affymetrix transcripts using Gene Symbol from the hta20transciprtcluster.db annotation. To match gene names listed with different aliases between two data sets, alternative gene names were searched on the National Center for Biotechnology Information (NCBI) Gene Database (https://www.ncbi.nlm.nih.gov/gene).

### Genotyping

We utilized the Illumina MEGA^EX^ array. After excluding poor quality SNPs, all SNPs had a call rate ≥ 0.95 and a replicate concordance 1.00 based on 39 QC replicate samples [11]. Eight well-studied SNPs in metabolic disease-related genes (Additional file 1: Table 1) were selected a priori to be tested for association with identified differentiated CpGs methylation by race/ethnicity.

**Table 1:** Characteristics of study participant women

Characteristics	All	Japanese Americans (n = 30)	European Americans (n = 28)
	Mean (SD) or N (%)	Mean (SD) or N (%)	Mean (SD) or N (%)
Age, years	63.4 (1.4)	63.4 (1.4)	63.5 (1.4)
Body mass Index, kg/m2	26.6 (4.6)	26.5 (4.7)	26.8 (4.5)
Total body fat mass, kg	27.0 (8.4)	25.5 (8.2)	28.6 (8.4)
Liver fat %, adjusted for total fat*	4.56 (0.51)	5.81 (0.98)	3.74 (0.55)
Smoking history			
Never	37 (64%)	22 (73%)	15 (54%)
Former	18 (31%)	7 (23%)	11 (39%)
Current	3 (5%)	1 (3%)	2 (7%)
Education			
12 years	5 (9%)	4 (13%)	1 (4%)
14 years	20 (34%)	13 (43%)	7 (25%)
16 years	17 (29%)	7 (23%)	10 (36%)
18 years	16 (28%)	6 (20%)	10 (36%)
Blood biomarkers			
C-reactive protein (CRP), mg/L	2.0 (3.3)	1.7 (3.6)	2.4 (2.9)
Macrophage inflammatory protein-1 beta (MIP1B), pg/mL	30.7 (12.9)	36.0 (13.9)	25.0 (8.8)
Triglycerides (TG), mg/dL	93.0 (75.6)	107.1 (95.9)	77.8 (41.5)
Insulin, mIU/L	8.3 (8.1)	10.5 (9.4)	6.0 (5.9)
Alanine Aminotransferase (ALT), U/L	31.8 (16.1)	34.4 (18.4)	29.0 (13.0)
Blood cell compositions			
CD8 + T cells	0.05 (0.04)	0.05 (0.03)	0.05 (0.04)
CD4 + T cells	0.15 (0.06)	0.15 (0.06)	0.16 (0.07)
Natural killer cells	0.09 (0.05)	0.10 (0.05)	0.08 (0.05)
B cells	0.06 (0.03)	0.08 (0.03)	0.05 (0.02)
Monocytes	0.09 (0.02)	0.09 (0.02)	0.10 (0.02)
Granulocytes	0.57 (0.08)	0.56 (0.08)	0.58 (0.07)

^*^For 46 participants (20 Japanese Americans, 26 European Americans) with MRI measurements

### Blood biomarkers

In the current analysis, we focused on fasting blood levels of 28 a priori selected biomarkers of lipid metabolism, insulin resistance, liver function and inflammation: specifically, lipid metabolism (high-density lipoprotein (HDL) and total cholesterol, triglycerides (TG)), insulin resistance (homeostatic model assessment for insulin resistance (HOMA-IR)), liver function (alanine and aspartate aminotransferases (ALT, AST), gamma-glutamyl transferase (GGT), cytokeratin 18 (CK18 M30 and M65), and sex hormone-binding globulin (SHBG)), and inflammation (component 3 (C3), high-sensitivity C-reactive protein (CRP), interleukinds and receptors (IL1R α, IL-6, IL6R, IL-10, IL-1β, IL-2, IL-4, IL-8, IL-5), tumor necrosis factor and receptors (TNFα, TNFR1, TNFR2), macrophage inflammatory protein 1 beta (MIP1B), monocyte chemoattractant protein 1 (MCP1), and tissue inhibitor of metalloproteinase 1 (TIMP1)). The priority for these biomarkers were based on their a priori importance for some (e.g., lipid and insulin markers that are used to define the metabolic syndrome) and based on their relevance after finding that a large proportion of differentially methylated CpGs was implicated in liver function and disease (e.g., liver-specific markers, SHBG that we found to be strongly inversely correlated with liver fat and NAFLD [42], and inflammation markers for their relevance in NAFLD and NASH). Analytical methods were reported previously [42]. All assays were performed in one or two batches on the same day, and the included blind duplicate quality control samples showed good reproducibility (coefficient of variation for all assays 2–20% [42]).

### Targeted replication of differentially methylated CpGs between JPA and EUA in the cancer genome atlas (TCGA) liver hepatocellular carcinoma (LIHC) data

We used the publicly available TCGA-LIHC database (https://portal.gdc.cancer.gov/projects/TCGA-LIHC) to examine the consistency with our differentially methylated CpGs between JPA and EUA. The Level 1 genome-wide DNA methylation data for the adjacent non-tumor tissues (24 samples from men, 16 samples from women) from 6 Asian LIHC cases (of no specified Asian subtype available) and 34 EUA cases were analyzed. The data were normalized using SWAN, and batch effects were removed by adjusting for batches before the data analysis.

### Statistical analyses

All statistical analyses were performed in R version 3.5.2, relevant Bioconductor version 3.7 packages, and the Partek Genomics Suite™ 6.6 (St. Louis, MO). To compare the descriptive characteristics of JPA versus EUA, Welch’s t tests were used for continuous traits and Fishers’ exact test for categorical traits. The total fat-adjusted liver fat values were obtained in general linear models (ANCOVA).

#### Identification and characterization of differentially methylated CpGs between JPA and EUA

CpGs differentially methylated by race/ethnicity (DM-CpGs) were identified in linear regression of the M-value for each CpG on race/ethnicity (JPA vs. EUA) (R Bioconductor, limma [43]). Significant CpGs (Bonferroni-correct *P* < 0.1) were visually examined for their ability to separate JPA from EUA by applying hierarchal clustering with Unweighted Pair Group Method with Arithmetic Mean (UPGMA; R, hclust). The CpGs were also examined in a volcano plot, showing the mean methylation difference in each for JPA versus EUA (Δβ; positive for hypermethylation and negative for hypomethylation in JPA vs. EUA) plotted against –log_10_[*P*-value].

Welch’s t test was used to compare the mean methylation of the DM-CpGs between JPA and EUA (*P* < 0.05 for significance) stratified by categories defined by the genomic location of the CpG or proximity to CpG islands. The genomic location (promoter, non-promoter, or intergenic) was defined according to the Illumina’s annotation file (https://support.illumina.com/content/dam/illumina-support/documents/downloads/productfiles/methylationepic/infinium-methylationepic-manifest-column-headings.pdf). Finally, the DM-CpGs were examined in Ingenuity Pathway Analysis (IPA) (Qiagen) to assess their likely functional involvement.

Separately, we compared the mean of 285,457 CpGs, as global methylation and the mean DNA methylation age (Hannum’s blood epigenetic clock [44]) between JPA and EUA using Welch’s t test.

#### Correlation of differentially methylated CpGs by race/ethnicity with gene expression and blood biomarkers

Pearson correlation was used for the association of DM-CpGs with their corresponding gene expression, using Benjamini and Hochberg False Discovery Rate (FDR) < 0.1 for significance. Spearman correlation was used to determine if the DM-CpGs were associated with each blood biomarker (FDR < 0.1 for significance).

#### Association between differentially methylated CpGs by race/ethnicity and SNPs

Pearson’s chi-square test was used to determine whether allele distribution differed by ethnicity for each SNP. To determine if genetic variants in metabolic disease-related SNPs were associated with DNA methylation at the DM-CpGs, ANOVA models were used. For each SNP, samples with missing data were removed, and an indicator variable was created that was 1 if the sample contained at least one risk allele. A simple ANOVA model was fit for each DM-CpG, with DNA methylation as the outcome and risk allele indicator as the covariate. FDR < 0.1 was used to determine significance.

#### Comparison of differentially methylated CpGs by race/ethnicity with TCGA liver tissue methylation

Once we found that the majority of the DM-genes between JPA and EUA were enriched in liver function/liver diseases, we utilized TCGA data to: (1) examine whether these CpGs were similarly differentially methylated in non-tumor liver tissue of Asians versus EUA and (2) examine whether these CpGs were differentially methylated between liver tumor and adjacent non-tumor tissue, in order to explore their potential involvement in liver tumorigenesis. To identify the CpGs differentially methylated between Asians (n = 6) versus EUA (n = 34) in their non-tumor liver tissue, we used three-way ANOVA with adjustment for age and sex. To identify the CpGs differentially methylated between hepatocellular carcinoma tumors and adjacent non-tumor tissues (n = 40 pairs), we performed two-way ANOVA with adjustment for pairs (FDR < 0.1 for significance).

## Results

### Participant characteristics

As reported previously [20], JPA and EUA women were all post-menopausal and had similar age and BMI distributions by study design, JPA had a higher level of total adiposity-adjusted liver fat compared to EUA (Table 1). JPA and EUA did not differ in smoking history, education, blood levels of CRP, TG, and ALT in Table 1. However, JPA had higher blood concentrations of MIP1B (36.0 pg/mL vs. 26.7 pg/mL in EUA; *P* = 0.006) and insulin (10.5 vs. 6.0 mIU/L; *P* = 0.03) compared to EUA.

### Identification of differentially methylated CpGs between JPA and EUA

**Fig. 1:**
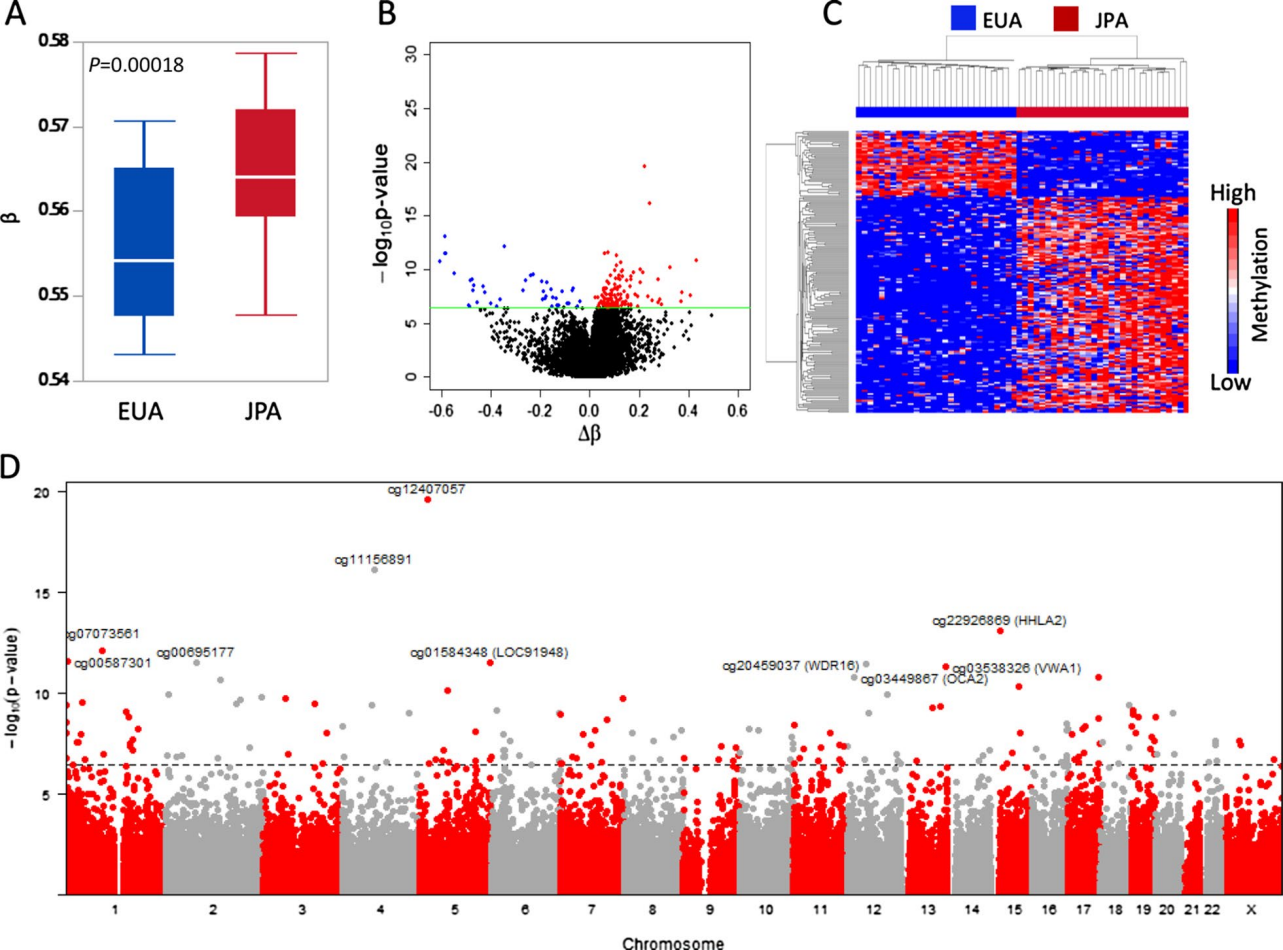
Global DNA methylation and 285,457 differentially methylated CpGs between JPA and EUA. **A** A box plot of global DNA methylation (mean methylation of 285,457 DM-CpGs) between JPA (red) and EUA (blue). **B** A volcano plot for all 285,457 CpGs showing differences in mean methylation (Δβ = Avg Methylation JPA—Avg Methylation EUA) (x-axis) and –log_10_*P*-value (y-axis). The green line indicates the Bonferroni-adjusted *P*-value < 0.1 cutoff. The red points correspond to significantly DM-CpGs that are hypermethylated in JPA relative to EUA, while the blue points correspond to DM-CpGs that are hypermethylated in EUA compared to JPA. **C** Hierarchal clustering of the 174 significantly DM-CpGs (rows) and samples (columns). Red corresponds to higher methylation, while blue corresponds to lower methylation. **D** A Manhattan plot showing the significance of association by chromosome, with the top 10 DM-CpGs by *P*-value labeled. The dotted line indicates the Bonferroni-adjusted *P*-value < 0.1 cutoff

When compared for the mean methylation level for all CpGs analyzed, a value that can be taken as a measure of global methylation, we observed a significantly higher mean methylation level in JPA (mean β = 0.565) compared to EUA (mean β = 0.556) (*P* = 0.00018) (Fig. 1A). For the blood epigenetic clock index, JPA had a higher mean DNA methylation age (64.19) than EUA (62.69), but the difference was not statistically significant (data not shown).

From the locus-specific analysis, we identified 730 differentially methylated CpGs (DM-CpGs) between JPA and EUA (Bonferroni *P* < 0.1), which were reduced to 174 DM-CpGs after adjusting for blood cell type composition: 160 of the 174 were found among the 730 (Additional file 2: Table 2). With respect to the cell proportions, JPA had significantly higher B cells and monocytes than EUA (*P* < 0.05), but did not differ for CD8 + T cells, CD4 + T cells, natural killer cells, and granulocytes (Table 1). None of the DM-CpGs were associated with the level of education.

**Fig. 2:**
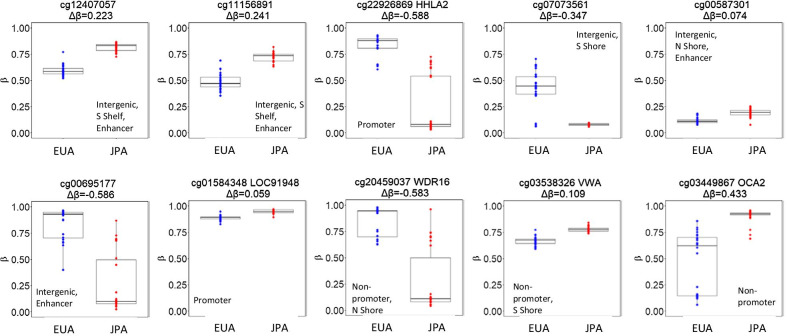
Dot plots of methylation (β-value) between JPA and EUA for the top ten significantly differentially methylated CpGs by *P*-value. All have *P*-value < 1.5e − 11 (FDR < 0.1). Blue points indicate EUA (n = 28), and red points indicate JPA (n = 30). Δβ is defined as average methylation in JPA—average methylation in EUA. Δβ > 0 corresponds to hypermethylation in JPA relative to EUA, while Δβ < 0 corresponds to hypermethylation in EUA relative to JPA

The majority of the DM-CpGs (n = 133, 76%) were hypermethylated in JPA compared to EUA, as shown in the volcano plot (Fig. 1B) and hierarchical clustering (Fig. 1C). The DM-CpGs were spread across all autosomes, as shown in the Manhattan plot (Fig. 1D). The top ten DM-CpGs based on statistical significance are presented in Fig. 2. Five CpGs were associated with genes *HHLA2*, *LOC91948*, *WDR16*, *VWA1*, and *OCA2*, as well as intergenic CpGs (cg12407057, cg11156891, cg07073561, cg00587301, and cg00695177). A full list of the 174 DM-CpGs and their genomic characteristics is provided in Additional file 3: Table 3.

### Characterization of differentially methylated CpGs between JPA and EUA

**Fig. 3:**
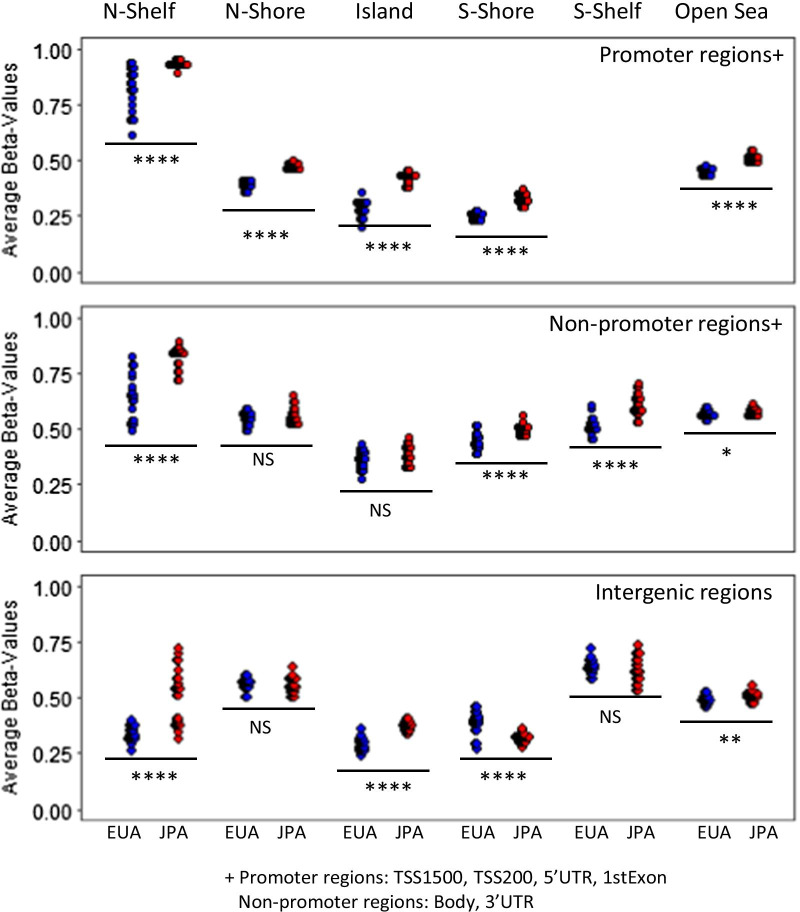
Methylation levels stratified by location of significant differentially methylated CpGs between JPA and EUA. Average methylation levels for each sample within CpG islands and surrounding regions are shown in promoters (top panel), non-promoters (middle panel), and intergenic regions (bottom panel). Blue corresponds to EUA, and red corresponds to JPA. Differences in average methylation for EUA and JPA in each region were tested using unpaired Welch’s t test (**** *P* < 0.0001, *** 0.0001 to 0.001, ** 0.001 to 0.01, * 0.01 to 0.05, NS not significant)

We compared the mean methylation of DM-CpGs for JPA compared to EUA within each functional location category. We found significantly more hypermethylation of DM-CpGs in JPA (36% vs. 15%) within the promoter region but did not detect differences within the non-promoter or intergenic region (Additional file 4: Table 4). Figure 3 shows a further comparison of mean methylation of the DM-CpGs in JPA versus EUA stratified by CpG island-related regions (CpG islands, North- or South-shelves, North- or South-shores, and open sea regions) within each genomic location (promoter, non-promoter, and intergenic). Across all CpG island-related regions, mean methylation varied by race/ethnicity the most in promoters. There was a large variation in the DM-CpG methylation positioned in N-shelf, among EUA for promoter or non-promoter location and among JPA for intergenic location. Mean methylation was significantly higher among JPA for 12 of the 15 regions, whereas it was higher among EUA for only S-shore in the intergenic region (*P* < 0.05).

### Correlation of differentially methylated CpGs between JPA and EUA with corresponding gene expression

**Fig. 4:**
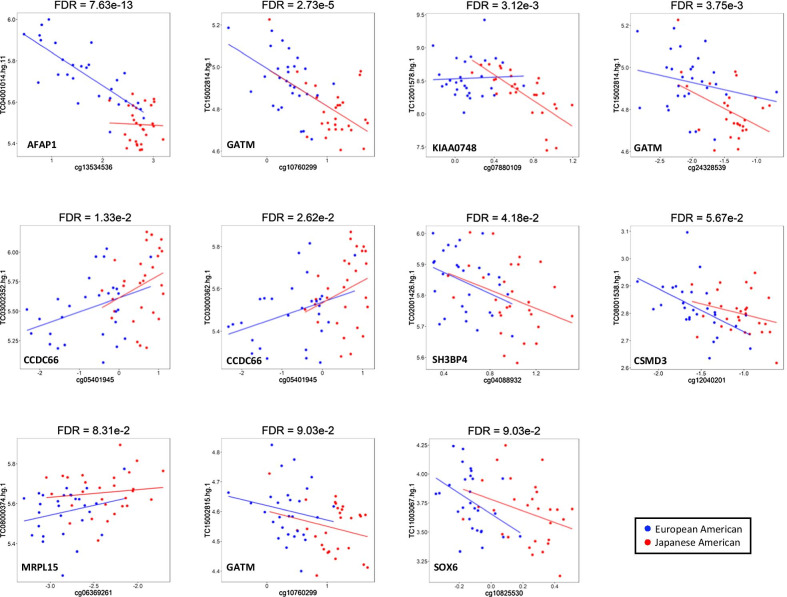
Scatter plots of transcript expression (log_2_) versus DNA methylation (M-value) with significant Pearson correlation by FDR < 0.1. The correlation FDR is indicated at the top of each plot. The blue points and lines correspond to EUA (n = 28), and the red points and lines correspond to JPA (n = 28)

Of the 174 DM-CpGs, 116 (67%) corresponded to 111 unique genes, resulting in 147 DM-CpG-transcript pairs (Additional file 5: Table 5). Eleven CpG-transcript pairs (9 unique CpGs and 10 unique transcripts) were significantly correlated at FDR < 0.1 (Fig. 4). *AFAP1*, *CSMD3*, *GATM* (3 transcripts), *KIAA0748*, *SH3BP4*, and *SOX6* were negatively correlated, and *CCDC66* (2 transcripts) and *MRPL15* were positively correlated. The strongest correlation was found for *AFAP1* (cg13534536-TC04001014.hg.1) (r =  − 0.83), driven by the correlation among EUA (r =  − 0.77), while no significant correlation was detected in JPA (r =  − 0.031). Also, the CpG-transcript correlation for *KIAA0748* was strong in JPA (r =  − 0.72) but not in EUA (r = 0.042).

### Potential biological roles of differentially methylated CpGs between JPA and EUA

We examined 102 of the 111 unique genes corresponding to the DM-CpGs in the IPA analysis and found that the majority of these genes (52, 51%) were functionally enriched in liver function/diseases, including liver inflammation, hyperplasia, proliferation, cirrhosis, and regeneration. These are summarized in Additional file 6: Table 6.

### Associations between differentially methylated CpGs between JPA and EUA and metabolic disease-related SNPs

We further explored the association between the DM-CpGs and eight previously reported metabolic disease-related SNPs (Additional file 1: Table 1). Among 174 DM-CpGs, 156 were significantly associated with rs7498665 (*SH2B1*), 3 with rs738409 (*PNPLA3*), and 49 with rs29941(*KCTD15*) at FDR < 0.1 (Additional file 7: Table 7). Some associations remained significant even after adjusting for race/ethnicity. These corresponded to 3 CpGs (cg22216157 [*PTPRN2*], cg24524099 [*PTPRN2*], and cg02903756 [*CASZ1*]) for rs738409 and 2 CpGs (cg07073561 [intergenic] and cg07863524 [*OR3A4*]) for rs29941.

### Correlation of differentially methylated CpGs between JPA and EUA with blood biomarkers of inflammation and metabolism

We further investigated the association of the methylation levels of 174 DM-CpGs with 28 blood biomarkers of inflammation, lipids, insulin resistance, and liver function. There were 183 significant CpG-biomarker associations, involving 149 CpGs (86% of DM-CpGs) and 5 biomarkers at FDR < 0.1: MIP1B (n = 146), HOMA-IR (n = 18), ALT (n = 1), IL-1β (n = 17), and IL-5 (n = 1) (Additional file 8: Table 8). Thirty-one CpGs were significantly correlated with multiple biomarkers, which were mostly associated with genes (n = 27; 87%). The majority of the CpG-biomarker associations (146/183, 80%) involved MIP1B, and top correlated CpGs were associated with genes, *SEPT9*, *HHAL2*, *OR3A4*, *NIPA1*,  and* PTPRN2*. None of the DM-CpGs were statistically associated with the other 23 biomarkers.

### Consistency of differentially methylated CpGs between JPA and EUA in TCGA-LIHC data

**Fig. 5:**
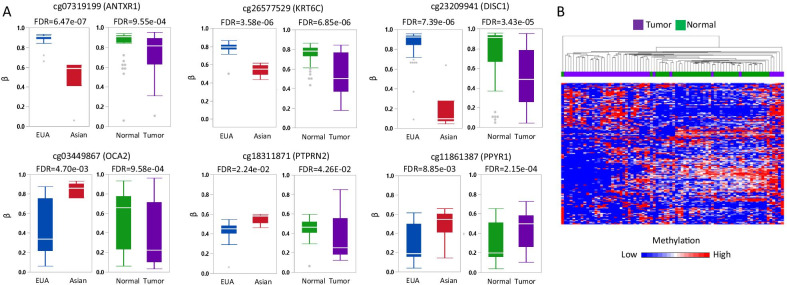
Targeted validation of differentially methylated CpGs between JPA and EUA in TCGA-LIHC dataset. **A** Example box plots of methylation (β-value) by race/ethnicity (left) and by tissue type (right). **B** A Manhattan plot showing clustering of 110 differentially methylated CpGs between tumor (purple) and adjacent non-tumor tissues (green) (40 pairs)

Given that the DM-CpGs between JPA and EUA are enriched in liver function and liver diseases/cancer, the 174 DM-CpGs were queried in the TCGA-LIHC data (Additional file 9: Table 9) to examine consistency in adjacent non-tumor tissues. We further explored the possible involvement of the DM-CpGs in tumorigenesis by comparing the CpGs between liver tumor and adjacent non-tumor tissue in the TCGA data. Thirty-eight CpGs (22%) were differentially methylated in the adjacent non-tumor tissues of Asian and EUA cases of hepatocellular carcinoma (FDR < 0.1), adjusted for age and sex, with 35 (92%) of them in a consistent racial/ethnic pattern as in our blood DNA analysis (Additional file 10: Table 10). For several example CpGs shown in Fig. 5A, the left panel depicts the Asian versus EUA difference. Of the 174 DM-CpGs, 110 CpGs (63%) were differentially methylated in paired tumor versus adjacent non-tumor tissues (pair-adjusted FDR < 0.1): the right panel of Fig. 5A depicts the tumor- non-tumor tissue difference. A heatmap of the 110 CpGs shows overall lower methylation of these CpGs in tumors compared to adjacent non-tumor tissues (Fig. 5B): the top five hypermethylated genes were *GLRX*, *WNT9B*, *SEPT9*, *KIAA00284*, and *PPYR1,* and the top five hypomethylated genes were *KIAA0748*, *PAEP*, *PCDH15*, *PTPRN2*, and *DUSP27* (Additional file 11: Table 11).

## Discussion

While some evidence points to racial/ethnic differences in blood DNA methylation and a postulated role for these differences in racial/ethnic health disparities [12, 45], data are still very limited on racial/ethnic comparisons and their biological implications. In this cross-sectional study, we found blood genome-wide differential methylation between JPA and EUA for 174 CpGs among generally healthy post-menopausal women. The majority of CpGs differentially methylated by race (DM-CpGs) (76%) were hypermethylated in JPA compared with EUA and highly enriched in promoter regions. The methylation levels of only a small subset of the DM-CpGs were positively or negatively correlated with their corresponding gene expression. Gene enrichment analysis for DM-CpGs revealed that the majority of the genes for these CpGs were involved in liver function and liver diseases, which may explain the substantially higher susceptibility of JPA to liver disease and liver cancer reported in this [20] and larger studies [6, 7]. Notably, most of the DM-CpGs were also associated with a blood biomarker of inflammation MIP1B. In the TCGA-LIHC (hepatocellular carcinoma) dataset, we confirmed some of the DM-CpGs in the TCGA non-tumor liver tissue of Asians compared to EUA. A large proportion of the DM-CpGs were also differentially methylated between liver tumor and non-tumor tissues.

Our study adds to the previous evidence for racial/ethnic differences in DNA methylation as reflecting underlying biological mechanisms possibly underlying some racial/ethnic health disparities.[17, 45–48]. Most past studies analyzed global DNA methylation, such as LINE-1 methylation, with inconsistent findings in small numbers of African or Hispanic ancestry [12]. In our study using a genome-wide methylation array, the mean methylation levels of all CpGs, a marker of global methylation, were significantly higher in JPA compared to EUA.

The majority of our DM-CpGs were involved in liver function and liver disease. Of the genes with DM-CpG-correlated gene expression, *AFAP1* and *KIAA0748* in particular were distinctly expressed in JPA versus EUA. *AFAP1*, found to be expressed at lower levels in JPA with hypermethylation of cg13534536 in this study, codes for actin filament associated protein 1, and its antisense RNA promotes liver tumor cell proliferation, indicative of a poor diagnosis [49]. *KIAA0748* is also known as *TESPA1,* and its deletion was detected in cirrhotic liver tissue [50]. Also differentially methylated and expressed in this study was *SOX6*, a transcription factor, acting as an activator of adipogenesis [51]. In the TCGA-LIHC dataset, we further found significantly differential methylation between tumor and adjacent non-tumor tissue for *AFAP1*, *KIAA0748*, and *SOX6*. Additional studies are needed to understand whether the liver diseases related to DM-genes contribute to the risk and progression of liver diseases.

Genetics may directly or indirectly explain an important part of the epigenetic differences by race/ethnicity. Here, we found 90% of the DM-CpGs between JPA and EUA to be significantly associated with rs7498665 (*SH2B1*). Also, we identified 3 CpGs to be associated with rs738409 (*PNPLA3*) and 49 for rs29941(*KCTD15*). S*H2B1* is a well-known metabolic regulator related to obesity and liver lipid metabolism [52, 53], and rs7498665 is associated with visceral fat [54]. *PNPLA3* genetic variant, rs738409, is a significant genetic risk factor for hepatic steatosis by accumulating high lipid droplets [55, 56]. Although the role of *KCTD15* is unclear in liver diseases, this gene is an obesity-related gene [57], and its genetic variation (rs29941) was significantly associated with weight changes [58] and fasting plasma glucose level [59]. As it is difficult in this small study for genetic polymorphisms to tease out independent SNP-CpG correlations from racial/ethnic differences in minor allele frequency, further study is warranted to investigate the role of DNA methylation in mediating racially/ethnically differential genetic susceptibility for metabolic diseases.

It is important to note that, in addition to the initial exclusion of SNPs associated probes in order to avoid potential risks of SNPs in the probe regions, we evaluated whether our 174 DM-CpGs overlap with any SNPs from 1000 Genomes available in the dbSNP database. We searched the chromosomal location of each CpG site and found that 21/174 (12.1%) overlap with the SNPs of 1000 Genomes. Of these 21, only 9 SNPs (9/174, 5.2%) had slightly different allele frequencies between East Asians and Europeans (e.g., C = 0.9990/T = 0.0010 vs C = 1.0000/T = 0.0000 for most cases). However, methylation levels at these 9 DM-CpGs had a continuous distribution, indicating no SNP effects on methylation at these CpGs.

Finally, we found eight DM-CpGs that were consistently associated with corresponding gene expression, important genetic variation (rs7498665, S*H2B1*), and systemic inflammation (MIP1B): these were cg12040201 in *CSMD3*, cg04088932 in *SH3BP4*, cg24328539 in *GATM*, cg05401945 in *CCDC66*, cg07880109 in *KIAA0748*, cg10760299 in *GATM*, cg10825530 in *SOX6*, and cg13534536 in *AFAP1*. *CSMD3* is considered a driver gene in liver cancer [60]. Although the biological function of *CSMD3* has not been fully understood, this gene is associated with alcohol exposure [61] and its genetic alteration is associated with morbid obesity [62]. *SH3BP4* is a potential tumor suppressor, and its methylation is related to impaired insulin signaling [63]. Elevated blood levels of proinflammatory cytokine MIP1B, also known as *CCL4,* are observed in liver inflammation and fibrosis [64, 65]. Future studies on a larger scale may be able to disentangle the genetic-epigenetic-phenotype associations by race/ethnicity.

This study has several strengths. It was nested within a long-term cohort providing the advantage of having well-characterized participants with various types of data. Our study integrated analysis of gene enrichment, gene expression, SNPs, blood biomarkers, and the TCGA. The consistency in findings across these analyses are supportive of the potential role of race/ethnicity-associated DNA methylation patterns in metabolism. However, this initial exploratory study had clear limitations, including a small sample size and the cross-sectional study design. While we had sufficient power to identify a number of significant DM-CpGs and detect their association with some biomarkers, larger multiethnic studies including both sexes are warranted to investigate racial/ethnic DNA methylation profiles more systematically. To identify potential drivers of DM-CpGs by race/ethnicity, additional studies for the effects of the environment, lifestyle, nutrition, and individual and contextual socioeconomic status on these associations should be performed with relevant health outcomes more carefully.

In conclusion, our findings provide supportive evidence that differential blood DNA methylation across racial/ethnic populations may represent epigenetic mechanisms underlying phenotype differences and disparities. Larger and more diverse studies are warranted to explore these relationships further.

## Supplementary Information

**Additional file 1: Supplementary Table 1.** Characteristics of metabolic disease-related SNPs.

**Additional file 2: Supplementary Table 2.** Significant DM-CpGs from analyses with and without adjusting for cell composition.

**Additional file 3: Supplementary Table 3.** Characteristics of the 174 DM-CpGs.

**Additional file 4: Supplementary Table 4.** Distribution of DM-CpGs across functional genomic regions.

**Additional file 5: Supplementary Table 5.** Estimated correlation between DM-CpGs and corresponding transcripts.

**Additional file 6: Supplementary Table 6** DM-CpG genes associated with liver function and liver tumorigenesis as identified by IPA.

**Additional file 7: Supplementary Table 7.** DM-CpGs association with metabolic disease-related SNPs.

**Additional file 8: Supplementary Table 8.** Correlation between DM-CpGs and blood biomarkers (FDR < 0.1).

**Additional file 9: Supplementary Table 9.** Characteristics of TCGA-LIHC samples (40 pairs of tumor and adjacent normal tissue).

**Additional file 10: Supplementary Table 10.** Among 174 DM-CpGs, differential methylation between Asian and EUA in normal tissue from TCGA-LIHC dataset (FDR < 0.1).

**Additional file 11: Supplementary Table 11.** Among 174 DM-CpGs, differential methylation between tumor (T) and adjacent normal (N) tissues from TCGA-LIHC dataset (FDR < 0.1).

## Data Availability

The datasets generated during and/or analyzed during the current study are available from the corresponding author and the MEC Research Committee on individual requests.

## Abbreviations

FDR: Benjamini and Hochberg False Discovery Rate
EUA: European Americans
CpG: CpG site (cytosine-guanine)
DM: Differentially methylated
JPA: Japanese Americans
MEC: The Multiethnic Cohort Study
SNP: Single nucleotide polymorphism
TCGA: The Cancer Genome Atlas
TSS: Transcription Start Site
UTR: Untranslated Region
